# Sortilin as a Biomarker for Cardiovascular Disease Revisited

**DOI:** 10.3389/fcvm.2021.652584

**Published:** 2021-04-16

**Authors:** Peter Loof Møller, Palle D. Rohde, Simon Winther, Peter Breining, Louise Nissen, Anders Nykjaer, Morten Bøttcher, Mette Nyegaard, Mads Kjolby

**Affiliations:** ^1^Department of Biomedicine, Aarhus University, Aarhus, Denmark; ^2^Department of Chemistry and Bioscience, Aalborg University, Aalborg, Denmark; ^3^Department of Cardiology, Gødstrup Hospital, NIDO, Herning, Denmark; ^4^PROMEMO and DANDRITE, Aarhus University, Aarhus, Denmark; ^5^Department of Health Science and Technology, Aalborg University, Aalborg, Denmark; ^6^Department of Clinical Pharmacology, Aarhus University Hospital, Aarhus, Denmark; ^7^Steno Diabetes Center Aarhus, Aarhus University Hospital, Aarhus, Denmark

**Keywords:** Dan-NICAD, OLINK, protein biomarkers, cardiovascular disease, pQTL, SORT1, sortilin

## Abstract

Genetic variants in the genomic region containing *SORT1* (encoding the protein sortilin) are strongly associated with cholesterol levels and the risk of coronary artery disease (CAD). Circulating sortilin has therefore been proposed as a potential biomarker for cardiovascular disease. Multiple studies have reported association between plasma sortilin levels and cardiovascular outcomes. However, the findings are not consistent across studies, and most studies have small sample sizes. The aim of this study was to evaluate sortilin as a biomarker for CAD in a well-characterized cohort with symptoms suggestive of CAD. In total, we enrolled 1,173 patients with suspected stable CAD referred to coronary computed tomography angiography. Sortilin was measured in plasma using two different technologies for quantifying circulating sortilin: a custom-made enzyme-linked immunosorbent assay (ELISA) and OLINK Cardiovascular Panel II. We found a relative poor correlation between the two methods (correlation coefficient = 0.21). In addition, genotyping and whole-genome sequencing were performed on all patients. By whole-genome regression analysis of sortilin levels measured with ELISA and OLINK, two independent *cis* protein quantitative trait loci (pQTL) on chromosome 1p13.3 were identified, with one of them being a well-established risk locus for CAD. Incorporating rare genetic variants from whole-genome sequence data did not identify any additional pQTLs for plasma sortilin. None of the traditional CAD risk factors, such as sex, age, smoking, and statin use, were associated with plasma sortilin levels. Furthermore, there was no association between circulating sortilin levels and coronary artery calcium score (CACS) or disease severity. Sortilin did not improve discrimination of obstructive CAD, when added to a clinical pretest probability (PTP) model for CAD. Overall, our results indicate that studies using different methodologies for measuring circulating sortilin should be compared with caution. In conclusion, the well-known *SORT1* risk locus for CAD is linked to lower sortilin levels in circulation, measured with ELISA; however, the effect sizes are too small for sortilin to be a useful biomarker for CAD in a clinical setting of low- to intermediate-risk chest-pain patients.

## Introduction

Coronary artery disease (CAD) is a common complex disease with a strong genetic contribution. A large number of genome-wide association studies (GWASs) have been conducted to identify risk loci and genes involved in the development of early onset myocardial infarction (1, 2), various aspects of atherosclerosis (3–6), and low-density lipoprotein (LDL) cholesterol levels (7–10). Since 2007, several new candidate genes affecting LDL cholesterol have been identified by GWAS, including the *SORT1* locus (7).

The *SORT1* locus on chromosome 1p13.3 is broad and comprises the genes *CELSR2-PSRC1-MYBPHL-SORT1*. The majority of LDL cholesterol–associated single-nucleotide polymorphisms (SNPs) are located in the intergenic region between *PSRC1* and *CELSR2*, and downstream of *SORT1* and *MYBPHL* (11). In addition, SNPs in the same genomic region have been found to convey changes in transcript levels of *SORT1, CELSR2*, and *PSRC1* (12). Fine-mapping studies have found that genetic variants in the major (risk) haplotype block containing rs599834, rs646776, rs629301, and rs12740374 are associated with lower *SORT1, CELSR2*, and *PSRC1* transcript levels in the human liver (12, 13). Thus, it is difficult to exactly pinpoint which gene(s) is(are) responsible for the risk of developing CAD, although it is believed that the CAD risk variant at 1p13.3 alters the levels of sortilin in the liver (12).

Apart from being strongly associated with plasma LDL cholesterol levels, the *SORT1* locus is also associated with CAD (3, 5, 6, 8, 14–17) and a wide range of different vascular subphenotypes including early-onset myocardial infarction (1, 2), coronary stenosis (5, 8), coronary artery calcification (6), abdominal aorta aneurysm (4), and aortic valve calcification (18). These associations are likely an effect of increased LDL cholesterol. Sortilin binds ApoB to facilitate hepatic export of very low-density lipoprotein in mouse models (19). Sortilin also augments PCSK9 secretion and increases the development of atherosclerosis (12, 19–21). Animal and cell studies demonstrate that sortilin also has a cholesterol-independent effect on atherosclerosis, where sortilin is involved in inflammation and calcification of the vessel wall (22, 23).

In addition to the liver, sortilin is also expressed in tissues that are involved in atherosclerosis development, including smooth muscle cells and macrophages (19, 22, 23). Notably, it is released from smooth muscle cells as a full-length receptor in extracellular vesicles (23), but also liberated from thrombocytes in a soluble form comprising the entire extracellular domain upon activation and degranulation (24). The biological functions of the vesicular and soluble receptor isoforms remain unknown.

Regardless of the source of varying levels of circulating sortilin, it has been proposed as a potential biomarker for cardiovascular diseases. Several studies have found association between plasma sortilin and various cardiovascular phenotypes, such as statin efficacy (25), PCSK9 levels (21, 25), CAD (24, 26), major adverse cardiovascular event risk (27), aortic calcification (27), and peripheral artery disease (28), but not with LDL cholesterol (29).

In this study, we measured plasma sortilin using two different methods in a cohort of low- to intermediate-risk patients (*n* = 1,173) with symptoms suggestive of CAD. All patients were referred to coronary computed tomography angiography (CTA) and are thus well-characterized with respect to coronary calcium score and clinical variables. Sortilin was measured using OLINK Cardiovascular Panel II (CVD II) and a high-sensitivity sortilin enzyme-linked immunosorbent assay (ELISA) developed in-house. The aim of this study was to evaluate sortilin as a biomarker for CAD in a well-characterized cohort by (1) identifying genetic variants associated with variation in plasma sortilin level, (2) analyzing the effect of common clinical risk factors for CAD on sortilin levels, (3) investigating the association of sortilin with coronary calcium score and CAD severity, and (4) testing if circulating sortilin could improve risk stratification of patients with symptoms of CAD.

## Materials and Methods

### Cohort Description

The study design of the Dan-NICAD 1 trial has been described previously (30, 31). In short, Dan-NICAD 1 is a prospective, multicenter, cross-sectional study including patients with low to intermediate pretest probability (PTP) of CAD, no previous history of CAD, and referred to coronary CTA because of symptoms suggestive of CAD, as evaluated by a cardiologist. Patients with signs of obstructive CAD by coronary CTA were referred to invasive coronary angiography (ICA) with fractional flow reserve (FFR) measurements to verify suspected obstructions. Obstructive CAD was defined as an FFR of <0.80 or as high-grade stenosis (>90% diameter stenosis) by visual assessment. Major exclusion criteria were age younger than 40 years and eGFR <40 mL/min.

Blood samples from all participants were drawn in connection with the coronary CTA. EDTA and heparin plasma were isolated and stored at −80°C. Of the full Dan-NICAD 1 trial comprising 1,675 patients, heparin plasma was available for 1,173 patients for ELISA. The OLINK CVD II panel was analyzed on the full cohort. To ensure head-to-head comparison of the two sortilin datasets, we included only the 1,173 consecutive patients, which had sortilin information from both methods. The study protocol was approved by the regional research ethical committee, and written consent was obtained.

### Coronary CTA and Invasive Angiography

All scans were performed on a 320-slice volume CT scanner (Aquilion One; Toshiba Medical Systems, Japan) in accordance with usual clinical guidelines. Imaging analysis included an Agatston coronary artery calcium score (CACS) and visual evaluation of luminal diameter stenosis estimation in each coronary segment. CAD severity at CTA was classified by diameter stenosis reduction in all segments with a reference vessel diameter >2 mm as no CAD: 0%; mild CAD: 1 to <30%; moderate CAD: >30 to <50%; and severe CAD: ≥50% diameter stenosis reduction.

Approximately 4 weeks after the coronary CTA, ICA was performed in patients with a suspected severe CAD at coronary CTA. ICA with FFR was performed in all segments with a 30–90% diameter stenosis at the ICA. Two-dimensional quantitative coronary analysis (QCA) was performed in an independent core laboratory (ClinFact, Leiden, the Netherlands). Hemodynamically obstructive CAD was defined as high-grade stenosis by visual assessment (>90% diameter stenosis) or by an ICA-FFR ≤0.80 in a vessel of ≥2.0-mm diameter. If ICA-FFR was indicated but not technically possible, QCA-based stenosis assessment was used with a cutoff of ≥50% diameter reduction. All other patients, including patients without severe CAD at coronary CTA, were classified as having no hemodynamically obstructive CAD.

### Protein Measurements

Levels of circulating sortilin were quantified using the OLINK platform and a high-sensitivity ELISA assay developed in-house. Plasma was collected at two different geographical locations (collection sites) in Denmark over a period of 2 years (30).

#### OLINK

EDTA plasma samples in 96-well matrix tubes were shipped to OLINK proteomics AB (Uppsala, Sweden) on dry ice for analysis using the proximity extension assay (PEA) technology. Levels of 92 proteins from the CVD II panel were measured, among them sortilin. Samples were replated and randomly distributed across assay plates at the OLINK analysis facility to allow for control of technical batch effects. The final assay read-out was Normalized Protein eXpression (NPX) values, which is an arbitrary unit on a log2 scale where high values correspond to higher protein levels. Using an internal extension control and an interpolate control, data quality was controlled and normalized. All assay validation data are available on the manufacturer's website (www.olink.com).

#### High-Sensitivity Custom-Made ELISA

Heparin plasma was thawed and measured using a custom-made ELISA against human soluble sortilin. In short, 96-well plate Nunc Maxisorp was coated with anti–human soluble sortilin [custom made against EC domain as described by Petersen et al. (32)] 1 μg per well in 55 mM NaHCO_3_ pH 9.8. The wells were subsequently blocked with 5% bovine serum albumin (BSA) (Sigma A7888) in phosphate-buffered saline (PBS) (pH 7.4). Washing was done in PBS-T (pH 7.4). Sample, internal control, and standard were incubated overnight at 4°C. Standard curve was made from 0.625 to 160 ng/mL of purified human soluble sortilin (21). The in-house generated detection antibody (MABN1792, Sigma) was diluted in PBS-T with 1% BSA to 1 μg/mL. One hundred microliters of the solution was added to each well and allowed to incubate at 37°C for 1 h. Finally, plates were incubated with peroxidase-conjugated goat anti–mouse immunoglobulin G (Dako) for 30 min at room temperature and rinsed five times before addition of *o*-phenylenediamine dihydrochloride (Dako) mixed with hydrogen peroxide. The reaction was stopped using 0.5 M sulfuric acid, and absorbance was subsequently measured at 490 nm.

To investigate if the anticoagulant used (heparin vs. EDTA) affected the measurable sortilin level, a subset of 44 Dan-NICAD individuals was randomly selected. In a paired design, sortilin was measured again in both EDTA and heparin plasma samples using the same approach as described above.

#### Normalization of Protein Levels

For EDTA plasma, variation linked to collection site (Supplementary Figure 1A) and OLINK analysis plate (Supplementary Figure 1B) was observed. For heparin plasma, variation linked to analysis plate (custom-made ELISA) was observed (Supplementary Figure 2). To account for the observed batch effects, the raw data were adjusted using linear regression. In this process, the sortilin measurements were first normalized using rank inverse (to obtain normality) and then adjusted for covariates [sex, age, age^2^, collection box ID, average protein level for OLINK CVD panels II and III (only for OLINK), and ELISA plate ID (only for ELISA)] using linear regression, and finally the adjusted values were once again normalized using rank inverse to address deviations from normality (Supplementary Figure 3).

### Genetic Data

#### Genotyping

Genomic DNA was isolated from frozen whole blood collected in 4 mL EDTA tubes. Genotyping was performed at deCODE Genetics using the Illumina Global Screening Array (Illumina, Inc., San Diego, CA). Quality control was performed in PLINK 1.9 (33) according to Marees et al. (34), accounting for individual and SNP missingness (–geno 0.02, –mind 0.02), sex discrepancy (–check-sex), minor allele frequency (–maf 0.05), and deviation from Hardy–Weinberg equilibrium (–hwe 1 × 10^−6^), resulting in 310,531 autosomal genetic variants. To account for cryptic relatedness, first- and second-degree relatives were identified based on identity by descent ($\hat{\pi }>0.2$) computed by PLINK 1.9 (one sample was removed). Multidimensional scaling by PLINK was applied to identify ancestral outliers (26 samples removed).

#### Imputation

Imputation was performed using the Michigan Imputation Server (35) based on 310,531 genotyped variants (reference panel HRC: r1.1.2016 GRCh37). SNPs with minor allele frequency below 0.05, missing genotype rate larger than 5%, deviation from Hardy–Weinberg equilibrium (*P* < 1 × 10^−6^), and imputation quality below 0.9 were removed, resulting in a total of 4,658,994 autosomal genetic variants, with positions annotated to genome build GRCh37.

#### Whole-Genome Sequencing

Whole-genome sequencing was performed using paired end sequencing with an average depth of 30 on the Illumina NovaSeq 6000 sequencing platform at deCODE genetics. Reads were processed in the quality control pipeline described by Jónsson et al. (36). A total of 52,783,013 autosomal variants were detected in the whole-genome sequencing data.

### Variance Explained by Common Genetic Variants

The proportion of variation in circulating sortilin that can be explained by common genetic variants, $h_{SNP}^{2}$, was estimated with genomic restrict maximum likelihood as implemented in the qgg package (37). First, the additive genomic relationship matrix was constructed as $G=\frac{\left({WW}^{\prime }\right)}{m}$, where ***W*** is the centered and scaled genotype matrix, and *m* is the number of genotypes (*m* = 4,658,994) (38). Next, variance components from the linear mixed model were estimated: ***y*** = ***Xb*** + ***Zg*** + ***e***, where ***y*** is a vector of phenotypes (i.e., either sortilin level by OLINK or ELISA), ***X*** and ***Z*** are design matrices linking the predicted fixed effects ($\hat{b}$) and the estimated random effects ($\hat{g}$) to the phenotype, and *e* is the remaining unexplained residuals. The included fixed effects were the same set of covariates as used in the genetic association analysis. The estimate of $h_{SNP}^{2}$ was determined as ${ĥ}_{SNP}^{2}=\frac{{\hat{\sigma }}_{g}^{2}}{{\hat{\sigma }}_{g}^{2}+{\hat{\sigma }}_{e}^{2}}$, where ${\hat{\sigma }}_{g}^{2}$ and ${\hat{\sigma }}_{e}^{2}$ are the estimated variance components of the random effects; $\hat{g}\sim N\left(0,G{\hat{\sigma }}_{g}^{2}\right)$, $\hat{e}\sim N\left(0,I{\hat{\sigma }}_{e}^{2}\right)$, where ***I*** is an identity matrix (assuming uncorrelated residuals) (37, 39).

### Protein Quantitative Trait Loci Analysis

To investigate if specific loci in the genome were associated with circulating sortilin levels, a protein quantitative trait loci (pQTL) analysis was performed, testing the association of 4,658,994 autosomal genetic variants with circulating sortilin levels using PLINK 2.0 (33). Prior to the analysis, genetic principal components were computed in PLINK using a set of high-quality autosomal genotyped variants (*m* = 310,531), to investigate if there remained any population stratification in the reduced set of individuals. Expectedly, as the Danish population is remarkably homogeneous (40), and we have removed ancestral outliers, we found no significant association between genetic principal components and sortilin levels; therefore, these were not included in pQTL analysis.

To test if additional pQTLs could be discovered by analyzing all variants identified from whole-genome sequencing, per-chromosome VCF files were converted to PLINK binary format followed by association analysis using PLINK 2.0 (33). In total 52,783,013 autosomal variants were analyzed for association with sortilin level, with a genome-wide significance threshold of *P* < 5 × 10^−8^. In addition to the single point analysis, a burden test of rare variants was conducted using the MUMMY pipeline (41), which builds on SMMAT, which performs a burden test, SKAT, SKAT-O, and a hybrid test combining burden testing and SKAT (42). In short, a GRM was calculated using GCTA (43), VCF files were converted to GDS files, and a list of all variant sites was created and filtered to include only variants with an allele frequency <0.05. Subsequently, two different strategies were applied. The first approach kept only severe variants (Ensembl most severe consequence stronger than missense) and grouped them by gene. The second approach included only coding variants weighted by CADD score.

### Association of Patient Characteristics With Sortilin Levels

To establish the effect of a wide range of patient characteristics on circulating plasma sortilin level, a multiple linear regression on sortilin levels was performed, including patient characteristics, cardiac symptoms, risk factors, biochemistry measurements, and results from coronary CTA (Table 1).

**Table 1:** Cohort description (*n* = 1,173).

**Patient characteristics (*n* = 1,173)**	
Sex, male	572 (48.8%)
Age (years)	57.2 ± 8.7
Body mass index (kg/m^2^)	26.8 ± 4.3
**Cardiac Symptoms**	
Type of chest pain	
Non-specific	187 (16.0%)
Atypical	418 (35.8%)
Typical	318 (27.2%)
Dyspnea	244 (20.9%)
**Risk Factors**	
Smoking	
Never	546 (46.7%)
Former	166 (14.2%)
Active	456 (39.0%)
Pack-year	11.7 ± 16.2
Diabetes mellitus	
Type 1	7 (0.6%)
Type 2	62 (5.3%)
Cholesterol-lowering treatment	270 (23.3%)
Hypertensive treatment	410 (35.2%)
**Biochemistry**	
Cholesterol (total, mmol/L)	5.5 ± 1.1
Glucose (fasting, mmol/L)	6.0 ± 1.2
Creatinine (mmol/L)	75.4 ± 14.2
eGFR (mL/min)	79.3 ± 10.4
**Imaging**	
Coronary artery calcium score group	
=0	588 (50.1%)
=1–399	455 (38.8%)
>400	130 (11.1%)
CAD severity group from coronary CTA	
No CAD	546 (46.8%)
Mild CAD	251 (21.5%)
Moderate CAD	90 (7.7%)
Severe CAD	279 (23.9%)
**Invasive coronary angiography**	
Obstructive CAD	117 (10.0%)

### Effect of Sortilin on Risk Stratification

PTP for CAD was calculated as previously described (44, 45) based on clinical risk factors including sex, age, angina symptoms, and number of risk factors (i.e., family history of early CAD, smoking, dyslipidemia, hypertension, and/or diabetes).

The prediction accuracy for CAD severity groupings and obstructive CAD was determined using PTP as baseline, which then was compared to models that included circulating plasma sortilin levels quantified by either OLINK or ELISA. The accuracy of prediction was assessed using fivefold cross-validation, in which the entire data first were divided in five folds. For CAD severity groups, we used a linear model for approximation, and for obstructive CAD, a logistic regression was applied. For both disease end points, the general model was ***y*****=*****Xb*****+*****e***, where the response variable was either CAD severity group or the binary disease outcome, ***X*** was the design matrix, and ***b*** was the vector of parameter estimates (i.e., coefficients of intercept, PTP, and sortilin by OLINK or ELISA), and ***e*** was the remaining residual. For each combination of four folds, the parameters were estimated (*t*) and used to predict the outcome of the individuals (*v*) in the fold not used in the training of the model parameter, ${\hat{y}}_{v}=X_{v}{\left(X_{t}^{\prime }X_{t}\right)}^{-1}X_{t}^{\prime }y_{t}$. The prediction accuracy of CAD severity group was determined using Pearson correlation coefficient between predicted value and observed CAD severity groups, and the accuracy of discriminating CAD cases was determined by the area under the receiver operating characteristic curve (AUC) as implemented in the qgg package (37).

## Results

### Patient Cohort

The cohort comprised 1,173 individuals in the age range 40 to 80 years, all referred to a CT scan for investigation for CAD, primarily due to chest pain. Of the 1,173 patients, 117 patients (10%) were diagnosed with obstructive CAD at ICA, with an equal distribution of disease cases across age classes (Supplementary Figure 4A). Overall, 6% of females and 15% of males in the cohort were diagnosed with obstructive CAD (Supplementary Figure 4B). Those diagnosed with obstructive CAD were on average older than those not diagnosed with CAD (Supplementary Figure 4C). The baseline characteristics of the cohort can be found in Table 1.

### Circulating Sortilin Levels

**Figure 1:**
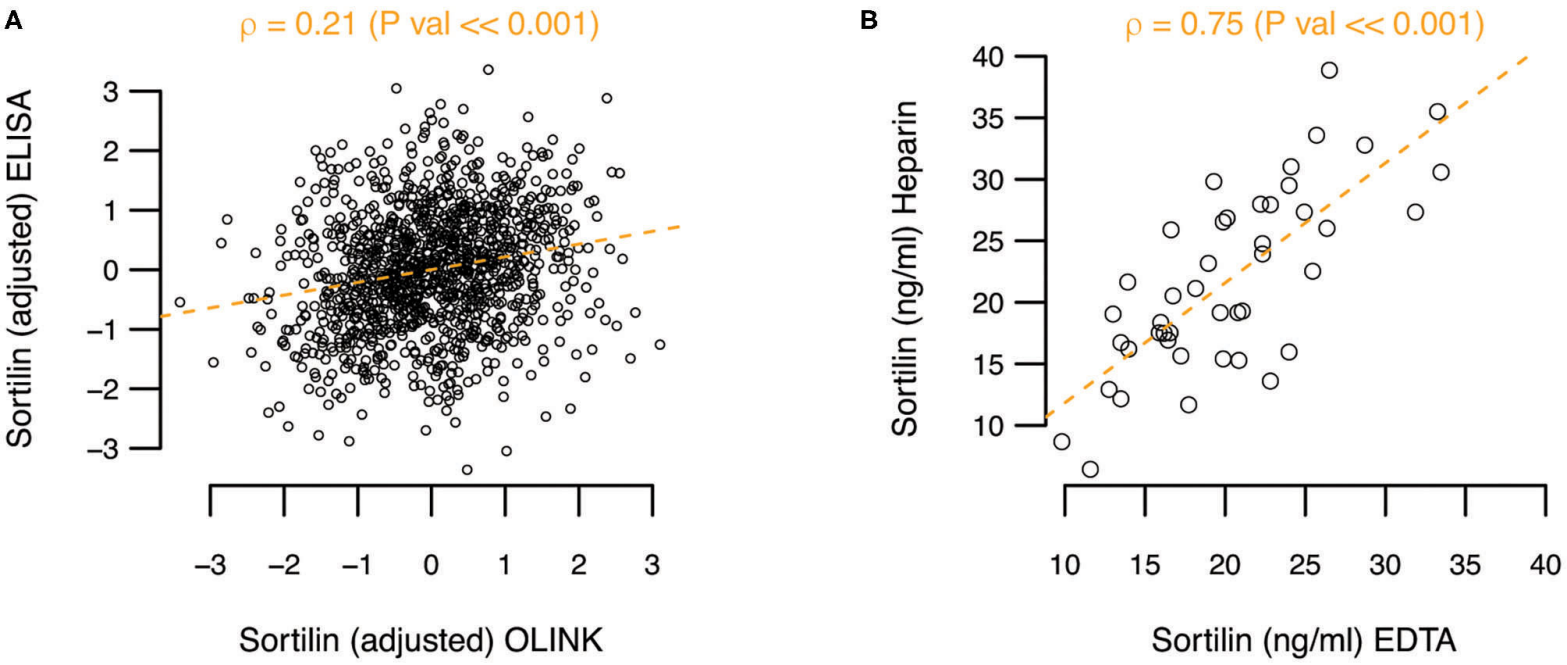
**(A)** Adjusted sortilin level from ELISA as function of adjusted sortilin level from OLINK. Pearson correlation coefficient (ρ) with significance level is noted above the plot. **(B)** Comparison of sortilin level measured with ELISA on paired heparin and EDTA plasma samples.

In a high-sensitivity ELISA assay, the mean sortilin plasma level was 30.99 ng/mL [95% confidence interval (CI) = 30.21–31.76 ng/mL] prior to adjustment and normalization. As OLINK only provides relative protein levels, the actual sortilin plasma levels between assays cannot be readily compared. However, when comparing the actual levels quantified by ELISA with the relative levels by OLINK, a rather poor correlation coefficient of ρ = 0.21 (*P* ≪ 0.001) was observed (Figure 1A). To explore if the anticoagulant used could explain part of the difference between the assays, we performed ELISA on paired heparin and EDTA plasma samples from 44 randomly selected Dan-NICAD individuals. While we find variation in the absolute level of sortilin between heparin and EDTA plasma for single individuals, we find on average no systematic difference between EDTA and heparin plasma. The correlation coefficient was ρ = 0.75, and slope = 0.97, indicating no systematic difference (Figure 1B).

### Genetic Analysis of Circulating Sortilin

To investigate how much of the variation observed in sortilin was explained by genetic variation, the SNP heritability (${ĥ}_{SNP}^{2}$) was estimated. None of the observed variation could be attributed to genetic variation as the estimates for sortilin measured with OLINK and ELISA were ${ĥ}_{SNP}^{2}$ = 1.4 × 10–9 (SE = 0.23) and ${ĥ}_{SNP}^{2}$ = 9.4 × 10–10 (SE = 0.23), respectively.

**Figure 2:**
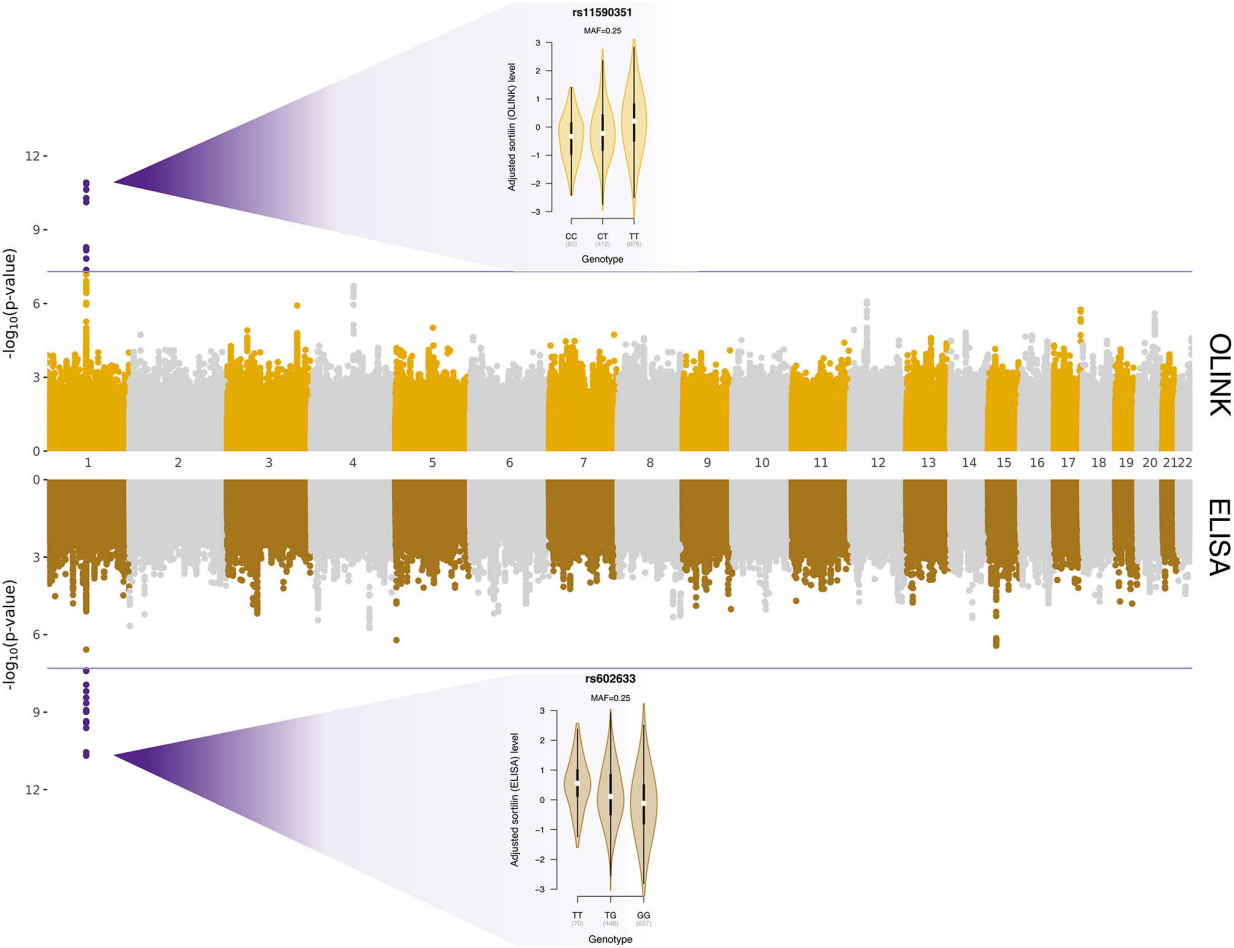
Protein quantitative trait loci for variation in sortilin level quantified with OLINK (top panel) and ELISA (bottom panel). The *x*-axis is chromosomal position, and the *y*-axes show the negative logarithm base-10 to the *P*-values from regression of circulating sortilin level on 4,658,994 autosomal genetic variants. The horizontal lines indicate the genome-wide significant threshold of 5 × 10^−8^, and points highlighted in purple are genetic variants surpassing this threshold. The two violin plots show the association between adjusted sortilin level and genotypes for the two identified *cis*-pQTLs.

**Figure 3:**
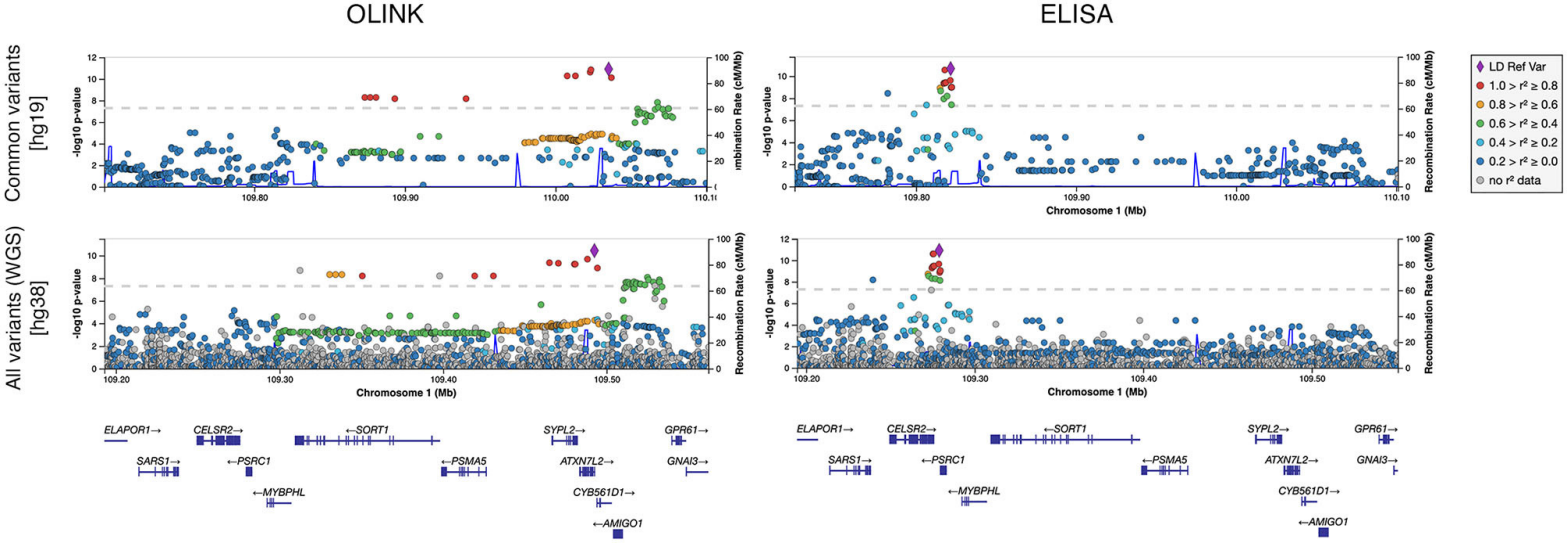
Regional plot of pQTLs identified for sortilin measured with OLINK and ELISA for common variants (MAF >0.05) and for all segregating variants identified by whole-genome sequencing (WGS).

Whole-genome regressions were performed to identify genetic variants associated with variation in sortilin levels. One pQTL for circulating sortilin measured with OLINK was identified on chromosome 1p13.3 with index SNP rs11590351 (*P* = 2.1 × 10^−11^) (Figure 2), and another pQTL using the ELISA data was identified also on chromosome 1p13.3 with the index SNP rs602633 (*P* = 2.1 × 10^−11^) (Figure 2). Interestingly, the two pQTLs are located on either side of *SORT1* and is not within the same LD block (Figure 3). The OLINK pQTL is positioned upstream of *SORT1*, with the minor allele (C, MAF = 0.25) being associated with decreased sortilin, whereas the ELISA pQTL is located downstream of *SORT1* where the minor allele (T, MAF = 0.25) is associated with increased sortilin (Figures 2, 3). The index variant for ELISA, rs602633, is also the index variant for CAD, with the major allele, G, being the risk variant (16).

Rare variant analysis identified 23,833 variants with more severe consequences than missense across 7,003 genes (median of two variants per gene) (Supplementary Figure 5). Limiting the analysis to coding variants weighted by CADD score identified 1,052,840 variants across 17,913 genes (median of 48 variants per gene). Neither of the two rare variants analyses found any significant associations between variants and circulating sortilin levels (Supplementary Figure 6). Analysis of all WGS variants individually did not provide any additional information on the molecular genetic basis of variation in circulating sortilin (Figure 3).

### Effect of Patient Characteristics on Sortilin Levels

**Figure 4:**
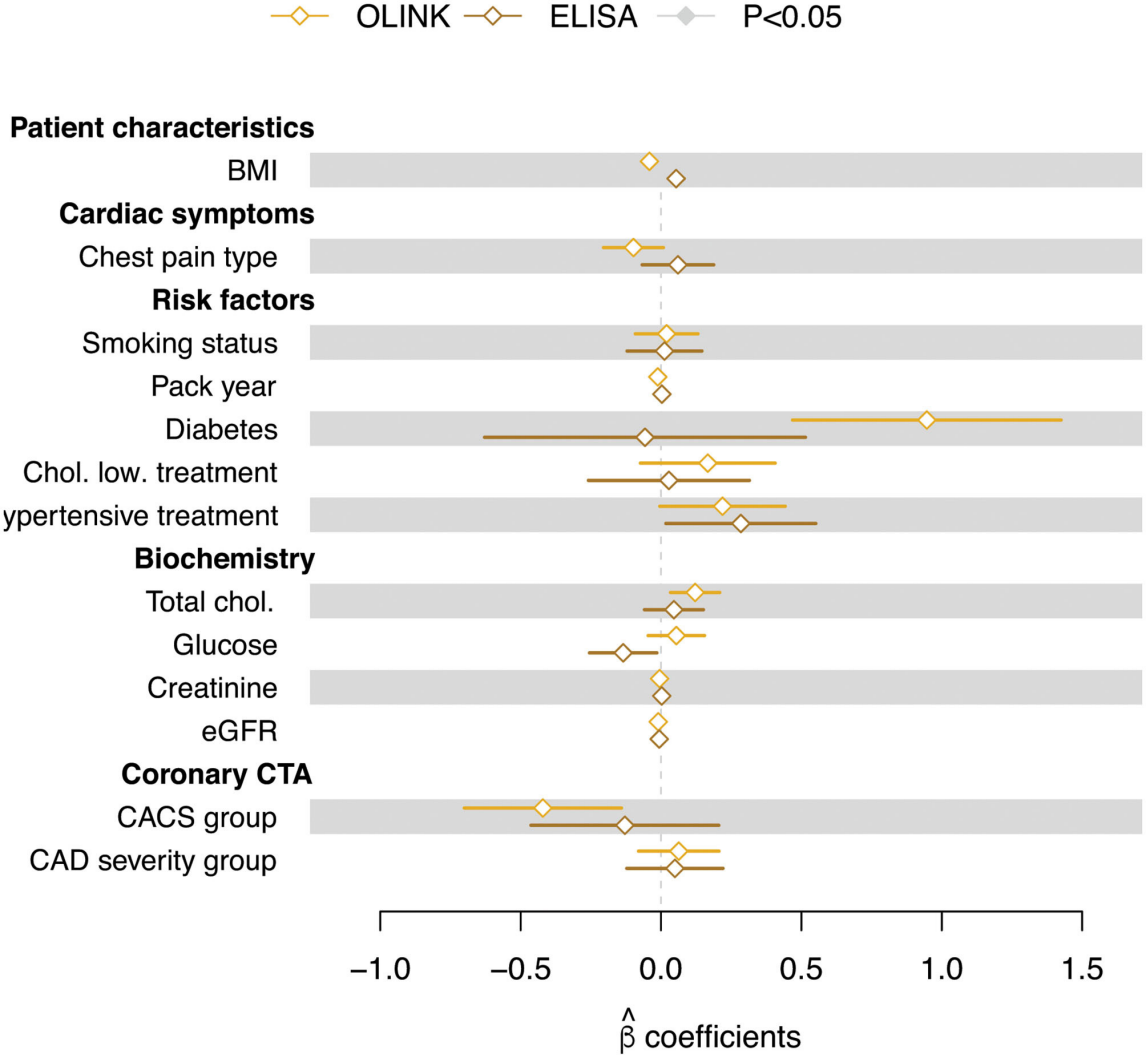
Regression coefficients (**β** estimates) from multiple linear regression of circulating sortilin (adjusted values) measured with OLINK and ELISA technologies. Error bars indicate the standard error on the estimate. Filled symbols indicate that the **β** estimates are significantly different from zero.

A multiple linear regression, testing for the effect of a number of patient characteristics, showed no significant effect of any of the tested variables (Figure 4). Importantly, no correlation was seen between sortilin levels and CACS (CACS group) or disease severity (CAD severity group) for either of the methods.

### Sortilin and Risk Stratification

**Figure 5:**
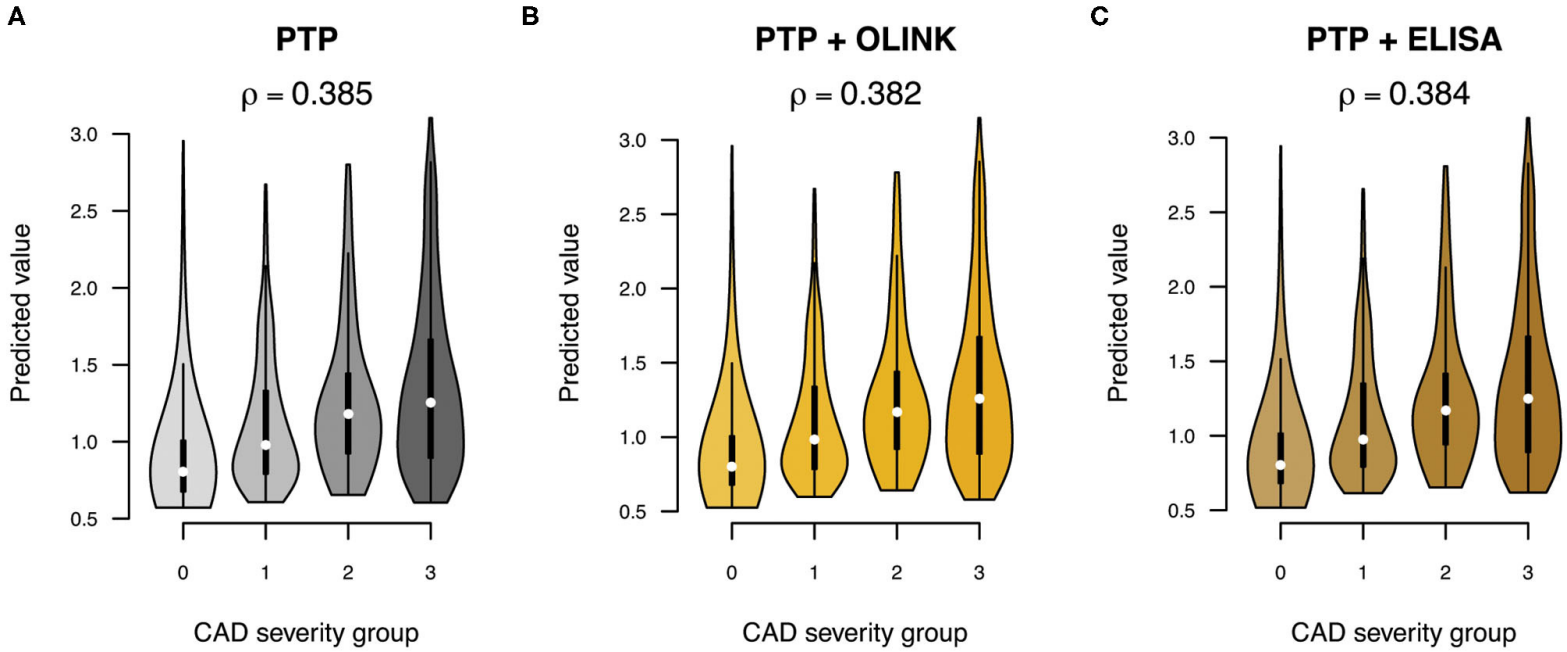
Prediction of CAD severity (0: no CAD, 1: mild CAD, 2: moderate CAD, 3: severe CAD) using **(A)** pretest probability (PTP) without and with sortilin measurements by **(B)** OLINK and **(C)** ELISA. The overall correlation (**ρ**) between predicted value and CAD severity group across the five validation sets is indicated above each plot.

**Figure 6:**
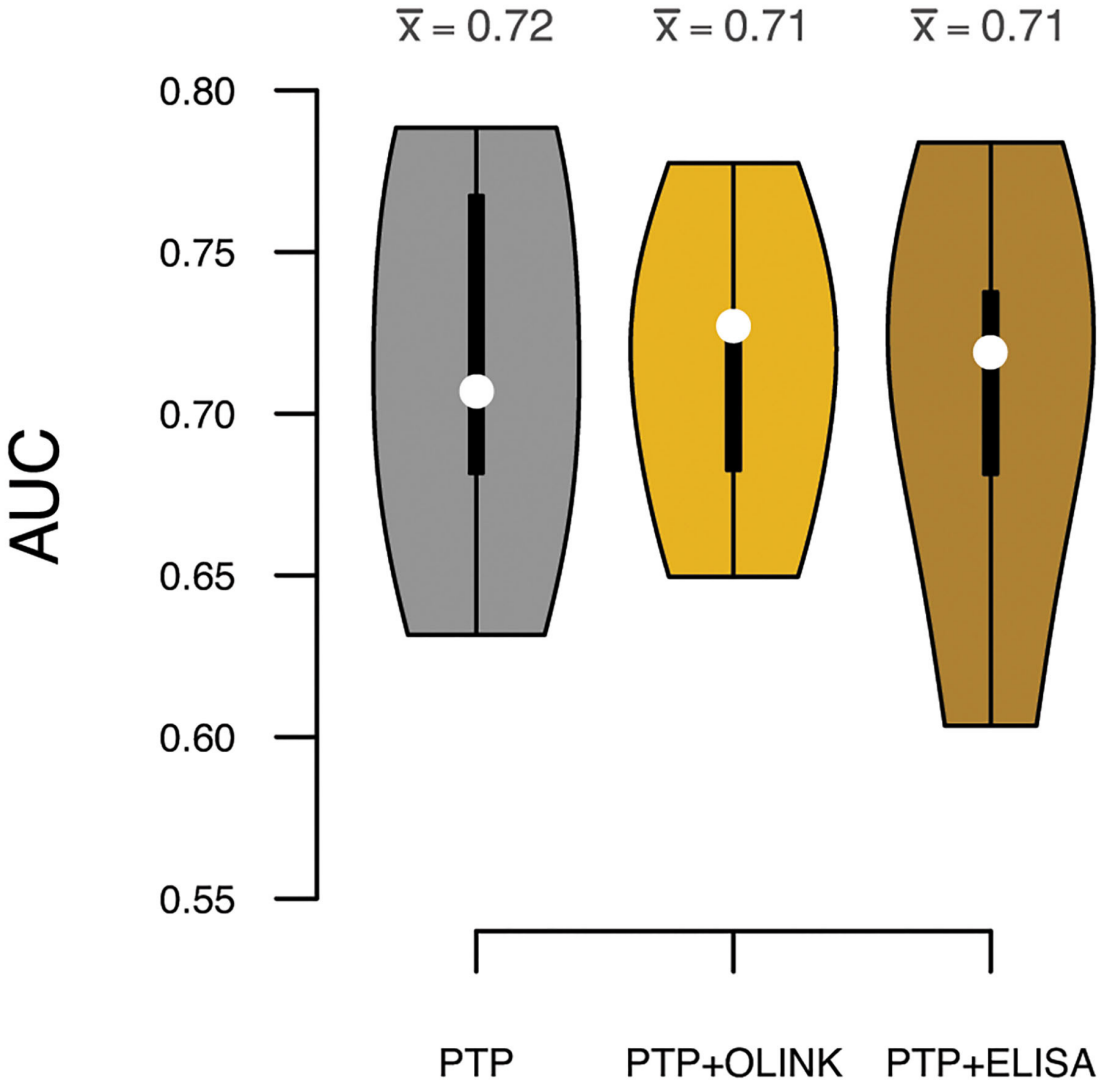
Prediction accuracies measured by area under the receiver operating characteristic curve (AUC) for discrimination of obstructive CAD. White dots indicate median AUC across the five validation sets, and the mean AUC ($\bar{x}$) for each model is indicated above each violin. Predictions were based on the pretest probability (PTP) with and without sortilin measurements by OLINK and ELISA.

To test if sortilin could provide an improvement in risk stratification of patients in addition to the traditional risk factors, we calculated the PTP for obstructive CAD with and without sortilin. The PTP calculated from traditional risk factors had, as expected, modest predictive power in stratifying patients in the four groups of increasing CAD severity (Pearson correlation ρ = 0.39, Figure 5). Including plasma sortilin levels from either OLINK or ELISA did not improve the predictive accuracy (Figure 5). The PTP had a mean accuracy for discriminating obstructive CAD evaluated with AUC of 0.72 (95% CI = 0.66–76) (Figure 6), which was marginally lower when including circulating sortilin (Figure 6).

In summary, we have found two independent *cis*-pQTLs for soluble sortilin, where the pQTL downstream of *SORT1* is colocalized with the CAD risk locus. However, we found no correlation between sortilin and CAD severity or coronary artery calcification, and no improvement in risk stratification for CAD, when adding sortilin on top of clinical risk factors, in patients suspected of CAD.

## Discussion

In this study, we investigated in a large well-described low- to intermediate-risk chest-pain cohort whether variation in plasma levels of soluble sortilin was under genetic influence and explored the added value of including soluble sortilin in risk stratification of patients with suspected CAD.

We identified two independent *cis*-pQTLs on chromosome 1p13.3 for circulating plasma sortilin measured with ELISA and OLINK (Figures 2, 3). For the pQTL downstream of *SORT1*, the lead SNP was rs602633; we found that the major G allele was associated with lower sortilin in plasma, which is in line with the well-known directional effect observed in the human liver (12), where the major G allele is associated with lower *SORT1* transcript levels. As the major G allele of rs602633 in large GWASs is strongly associated with higher LDL cholesterol (16, 46), one would expect lower sortilin levels in the plasma in individuals with CAD.

The few published studies investigating sortilin levels in smaller CAD study populations find either a positive or negative correlation (24, 26). In a cross-sectional study with 31 CAD patients and 116 healthy controls, Oh et al. (26) found higher levels of plasma sortilin in patients with CAD. In contrast, a study by Ogawa et al. (24) with 91 CAD patients and 189 healthy controls found lower plasma sortilin in CAD patients. Both studies used ELISA to detect sortilin. In the Dan-NICAD cohort, we were not able to detect any significant association between circulating sortilin and CAD.

We found a relatively low correlation between sortilin levels measured using two different methods applied. The reason for this can be assay or sample dependent. To our knowledge, there have not been any published studies comparing measurement of sortilin in ELISA (gold standard) and OLINK or other discovery platforms head-to-head. Differences between ELISA of specific analytes and various omics platforms, such as SOMAscan, Luminex xMAP, Myriad RBM, and OLINK, have been widely observed (47). Raffield et al. compared 20 different proteins measured using the Luminex xMAP and the SOMAscan platform and found that the correlations range from −0.13 to 0.97 between proteins, and nearly half had a correlation coefficient of <0.5. Furthermore, Raffield et al. compared pQTL analyses on the same cohort (SPIROMICS and COPDGene) measuring the same proteins but with different methods (Myriad RBM or OLINK and SOMAscan) (47) and found this affected the ability to detect *cis*-pQTLs. Additionally, there was very low correlation between proteins measured with the various methods in 20–40% of cases. It is clear that many of the high-throughput discovery methods such as OLINK, Myriad RBM, and SOMAscan need further validation by more conventional methods with true quantification, such as ELISA. In the OLINK assay, heparin plasma was used, whereas in the high-sensitivity ELISA assay, EDTA plasma was used. Thrombocytes contain sortilin (24), and neither heparin nor EDTA completely inhibited degranulation of thrombocytes (48). The degranulation is most pronounced in serum (24). Therefore, the anticoagulant may have affected the levels of sortilin in plasma. We did, however, not find on average any systematic difference between EDTA and heparin plasma when measuring paired EDTA and heparin samples with ELISA (Figure 1B).

Although both assays measure plasma sortilin, the detection is by different means. OLINK relies on the PEA technology, which uses a matched pair of monoclonal antibodies, each tagged with a unique DNA sequence. When the two antibodies bind sortilin, the DNA tags get into proximity and can hybridize, and a quantitative polymerase chain reaction can be performed. The output of OLINK is therefore relative NPX values on a log2 scale. In contrast, the ELISA method relies on coating of plates with polyclonal antibody, recognizing several isoforms in sortilin. Detection is with a monoclonal antibody and a secondary horseradish peroxidase–coupled antibody. Each plate includes a standard curve with samples of known sortilin concentration, allowing for absolute quantification of sortilin levels. As both techniques are based on antibody detection, one can speculate that posttranslational modifications of sortilin, such as glycosylation or carbamoylation, may have an impact on the detection efficacy, as this can mask epitopes, and may impact the assays differently. To determine if posttranslational modifications are responsible for the low correlation between the two assays, it will require further analyses including Western blotting and mass spectometry, which is beyond the scope of this study.

The variation found in sortilin levels in human plasma was not explained by traditional risk factors for CAD. Furthermore, the GWAS of plasma sortilin levels identified two independent *cis*-pQTLs (one for each of the platform used) at 1p13.3, where *SORT1* resides. The rare variant analyses did not detect any additional pQTLs. The source of sortilin in plasma may be a result of shedding from various tissues as sortilin is ubiquitously expressed (19, 22, 49–51). Sortilin may also be present in plasma as extracellular vesicles secreted by smooth muscle cells (23) or can be released from degranulated thrombocytes (24). The main source of variation is currently unknown, and the genetic contribution is low.

The lead pQTL SNPs for OLINK and ELISA sortilin were located upstream and downstream of *SORT1*, respectively. As the two loci were genetically uncoupled, this could indicate the existence of at least two regulatory sequences upstream and downstream of the *SORT1* gene. Two large pQTL studies on OLINK proteins have been published (52, 53). One study investigated 1,992 individuals on OLINK CVD II panel (includes sortilin) on EDTA plasma. Recently, a second large pQTL study was published comprising 1,328 individuals on the CVD II panel on serum samples. Bretherick et al. (53), were able to detect a *cis*-pQTL for sortilin in plasma at rs7528419, which is in strong LD with our lead SNP. Gilly et al. (52), however, were not able to detect any pQTL for serum sortilin. It is well-known that sortilin (among other proteins) can be released from degranulating thrombocytes, especially in serum (24). This is likely why a pQTL is not detected by Gilly et al. (52). Further evidence for the *cis*-pQTL downstream of *SORT1* for plasma sortilin, i.e., rs646776 and rs12740374, was found in other studies based on Luminex platform (54) and SOMAscan (55). None of the studies validated the findings using more than one platform.

As elevated total cholesterol and diabetes often are highly correlated, it was not surprising that we observed a tendency of non-zero regression estimates for both total cholesterol level and diabetes status. That diabetes status and elevated cholesterol showed a tendency of being associated with increased sortilin level in plasma is in agreement with sortilin being involved in glucose homeostasis (56, 57) and that diabetes subjects have previously been shown to have elevated sortilin levels (28). However, this positive correlation was not identified with ELISA. Diabetes does involve random glycosylation of proteins, and it is possible that this influences the detection efficacy in the above assays. Although obesity is a major risk factor of CAD, and sortilin is highly expressed in adipocytes (58), plasma sortilin levels weakly and negatively correlate (OLINK) with body mass index in our current study. No correlation was detected in ELISA. Additionally, even though plasma sortilin and liver sortilin seem to be regulated in the same direction by the major allele, this is not true for fat tissue (12). As there are many possible sources of sortilin in plasma, it does seem that fat is not a major source of sortilin.

The ability to accurately stratify patients with symptoms of CAD is challenging, and most stratification models rely on classical risk factors, such as age, sex, and symptoms (59), and degree of artery calcification (45). With the recent developments in omic's technologies, much effort has been put into combining traditional risk factors with omic's derived risk scores, which has so far shown some increased accuracy in discriminating healthy from affected patients (60–62). We used the PTP with and without sortilin measurements to investigate whether adding sortilin improved the accuracy of risk stratification. We did not find any improvement in risk prediction by including sortilin combined with the traditional risk factors (Figures 5, 6). This is in line with Bom et al. (63), who in a cohort of 203 patients with suspected CAD utilized machine learning to identify two protein signatures predicting high-risk plaques and absence of CAD, respectively. Neither protein signature included sortilin.

In conclusion, we have identified two *cis*-pQTLs for plasma sortilin at 1p13.3, where one of the pQTLs is a strong risk variant for CAD, with the risk allele for CAD driving lower levels of soluble sortilin in plasma, which is the same direction as the *SORT1* transcript in the human liver. We find no association of plasma sortilin with CAD severity or coronary artery calcification, and sortilin did not improve existing risk classification in a clinical setting. Overall, in a well-characterized cohort of patients, sortilin was not a useful biomarker for CAD.

## Data Availability Statement

The summary statistics for the pQTL analyses are available through GWAS catalog. The genotypes and WGS presented in this article are not readily available because of the sensitive nature of this data. Requests to access the datasets should be directed to Mette Nyegaard, nyegaard@biomed.au.dk.

## Ethics Statement

The studies involving human participants were reviewed and approved by The Danish Data Protection Agency (Case no. 1-16-02-345-14) and The Central Denmark Region Committee on Health Research Ethics (Case no. 1-10-72-190-14). All patients providing informed written consent. The study was registered at ClinicalTrials.gov (Identifier: NCT02264717). The patients/participants provided their written informed consent to participate in this study.

## Author Contributions

MK and MN conceptualized the study. MB, SW, and LN were responsible for the patient recruitment, phenotyping, and image analysis of patients. MN and PLM were responsible for building the biobank containing biological material from the Dan-NICAD 1 trial. MK and AN performed the ELISA analysis. MN, PLM and PDR analyzed the OLINK data. PDR processed, imputed, and performed the pQTL analysis on variants from genotyping. PLM processed and performed pQTL analysis on variants from whole genome sequencing. PLM and PDR carried out the statistical analysis. MN, MK, PDR and PLM drafted the first version of the manuscript. All authors read, provided comments and approved the final manuscript.

## Conflict of Interest

MK and AN has a patent (EP 3 666 281 A1) regarding using fragments of sortilin for treatment of insulin resistance. The remaining authors declare that the research was conducted in the absence of any commercial or financial relationships that could be construed as a potential conflict of interest.

## References

[1] Kathiresan S Voight BF Purcell S Musunuru K Ardissino D Mannucci PM. Genome-wide association of early-onset myocardial infarction with single nucleotide polymorphisms and copy number variants. Nat Genet. (2009) 41:334–41. 10.1038/ng.327 19198609 PMC2681011

[2] Wang AZ Li L Zhang B Shen GQ Wang QK. Association of SNP rs17465637 on chromosome 1q41 and rs599839 on 1p13.3 with cyocardial infarction in an American caucasian population. Ann Hum Genet. (2011) 75:475–82. 10.1111/j.1469-1809.2011.00646.x 21463265 PMC3115468

[3] Arvind P Nair J Jambunathan S Kakkar VV Shanker J. CELSR2-PSRC1-SORT1 gene expression and association with coronary artery disease and plasma lipid levels in an Asian Indian cohort. J Cardiol. (2014) 64:339–46. 10.1016/j.jjcc.2014.02.012 24674750

[4] Jones GT Bown MJ Gretarsdottir S Romaine SPR Helgadottir A Yu G. A sequence variant associated with sortilin-1 (*SORT1*) on 1p13.3 is independently associated with abdominal aortic aneurysm. Hum Mol Genet. (2013) 22:2941–7. 10.1093/hmg/ddt141 23535823 PMC3690970

[5] Muendlein A Geller-Rhomberg S Saely CH Winder T Sonderegger G Rein P. Significant impact of chromosomal locus 1p13.3 on serum LDL cholesterol and on angiographically characterized coronary atherosclerosis. Atherosclerosis. (2009) 206:494–9. 10.1016/j.atherosclerosis.2009.02.040 19380133

[6] Odonnell CJ Kavousi M Smith AV Kardia SLR Feitosa MF Hwang SJ. Genome-wide association study for coronary artery calcification with follow-up in myocardial infarction. Circulation. (2011) 124:2855–64. 10.1161/CIRCULATIONAHA.110.974899 22144573 PMC3397173

[7] Teslovich TM Musunuru K Smith AV Edmondson AC Stylianou IM Koseki M. Biological, clinical and population relevance of 95 loci for blood lipids. Nature. (2010) 466:707–13. 10.1038/nature09270 20686565 PMC3039276

[8] Samani NJ Erdmann J Hall AS Hengstenberg C Mangino M Mayer B. Genomewide association analysis of coronary artery disease. N Engl J Med. (2007) 357:443–53. 10.1056/NEJMoa072366 17634449 PMC2719290

[9] Kathiresan S Melander O Guiducci C Surti A Burtt NP Rieder MJ. Six new loci associated with blood low-density lipoprotein cholesterol, high-density lipoprotein cholesterol or triglycerides in humans. Nat Genet. (2008) 40:189–97. 10.1038/ng.75 18193044 PMC2682493

[10] Shungin D Deng WQ Varga TV. Luan J Mihailov E Metspalu A. Ranking and characterization of established BMI and lipid associated loci as candidates for gene-environment interactions. PLoS Genet. (2017) 13:e1006812. 10.1371/journal.pgen.1006812 28614350 PMC5489225

[11] Kjolby M Nielsen MS Petersen CM. Sortilin, encoded by the cardiovascular risk gene *SORT1*, and its suggested functions in cardiovascular disease. Curr Atheroscler Rep. (2015) 17:496. 10.1007/s11883-015-0496-7 25702058

[12] Musunuru K Strong A Frank-Kamenetsky M Lee NE Ahfeldt T Sachs KV. From noncoding variant to phenotype via *SORT1* at the 1p13 cholesterol locus. Nature. (2010) 466:714–9. 10.1038/nature09266 20686566 PMC3062476

[13] Schadt EE Molony C Chudin E Hao K Yang X Lum PY. Mapping the genetic architecture of gene expression in human liver. PLoS Biol. (2008) 6:1020–32. 10.1371/journal.pbio.0060107 18462017 PMC2365981

[14] Wallace C Newhouse SJ Braund P Zhang F Tobin M Falchi M. Genome-wide association study identifies genes for biomarkers of cardiovascular disease: serum urate and dyslipidemia. Am J Hum Genet. (2008) 82:139–49. 10.1016/j.ajhg.2007.11.001 18179892 PMC2253977

[15] Lee JY Lee BS Shin DJ Woo Park K Shin YA Joong Kim K. A genome-wide association study of a coronary artery disease risk variant. J Hum Genet. (2013) 58:120–6. 10.1038/jhg.2012.124 23364394

[16] Van Der Harst P Verweij N. Identification of 64 novel genetic loci provides an expanded view on the genetic architecture of coronary artery disease. Circ Res. (2018) 122:433–43. 10.1161/CIRCRESAHA.117.312086 29212778 PMC5805277

[17] Erdmann J Kessler T Munoz Venegas L Schunkert H. A decade of genome-wide association studies for coronary artery disease: the challenges ahead. Cardiovasc Res. (2018) 114:1241–57. 10.1093/cvr/cvy084 29617720

[18] Smith JG Luk K Schulz CA Engert JC Do R Hindy G. Association of low-density lipoprotein cholesterol-related genetic variants with aortic valve calcium and incident aortic stenosis. JAMA. (2014) 312:1764–71. 10.1001/jama.2014.13959 25344734 PMC4280258

[19] Kjolby M Andersen OM Breiderhoff T Fjorback AW Pedersen KM Madsen P. Sort1, encoded by the cardiovascular risk locus 1p13.3, is a regulator of hepatic lipoprotein export. Cell Metab. (2010) 12:213–23. 10.1016/j.cmet.2010.08.006 20816088

[20] Strong A Ding Q Edmondson AC Millar JS Sachs KV Li X. Hepatic sortilin regulates both apolipoprotein B secretion and LDL catabolism. J Clin Invest. (2012) 122:2807–16. 10.1172/JCI63563 22751103 PMC3408750

[21] Gustafsen C Kjolby M Nyegaard M Mattheisen M Lundhede J Buttenschøn H. The hypercholesterolemia-risk gene *SORT1* facilitates PCSK9 secretion. Cell Metab. (2014) 19:310–8. 10.1016/j.cmet.2013.12.006 24506872

[22] Mortensen MB Kjolby M Gunnersen S Larsen JV Palmfeldt J Falk E. Targeting sortilin in immune cells reduces proinflammatory cytokines and atherosclerosis. J Clin Invest. (2014) 124:5317–22. 10.1172/JCI76002 25401472 PMC4348947

[23] Goettsch C Hutcheson JD Aikawa M Iwata H Pham T Nykjaer A. Sortilin mediates vascular calcification via its recruitment into extracellular vesicles. J Clin Invest. (2016) 126:1323–36. 10.1172/JCI80851 26950419 PMC4811143

[24] Ogawa K Ueno T Iwasaki T Kujiraoka T Ishihara M Kunimoto S. Soluble sortilin is released by activated platelets and its circulating levels are associated with cardiovascular risk factors. Atherosclerosis. (2016) 249:110–5. 10.1016/j.atherosclerosis.2016.03.041 27085161

[25] Hu D Yang Y Peng D quan. Increased sortilin and its independent effect on circulating proprotein convertase subtilisin/kexin type 9 (PCSK9) in statin-naive patients with coronary artery disease. Int J Cardiol. (2017) 227:61–5. 10.1016/j.ijcard.2016.11.064 27846466

[26] Oh TJ Ahn CH Kim BR Kim KM Moon JH Lim S. Circulating sortilin level as a potential biomarker for coronary atherosclerosis and diabetes mellitus. Cardiovasc Diabetol. (2017) 16:92. 10.1186/s12933-017-0568-9 28728579 PMC5520342

[27] Goettsch C Iwata H Hutcheson JD O'Donnell CJ Chapurlat R Cook NR. Serum sortilin associates with aortic calcification and cardiovascular risk in men. Arterioscler Thromb Vasc Biol. (2017) 37:1005–11. 10.1161/ATVBAHA.116.308932 28279970 PMC5407935

[28] Biscetti F Bonadia N Santini F Angelini F Nardella E Pitocco D. Sortilin levels are associated with peripheral arterial disease in type 2 diabetic subjects. Cardiovasc Diabetol. (2019) 18:5. 10.1186/s12933-019-0805-5 30634965 PMC6329108

[29] Nozue T Hattori H Ogawa K Kujiraoka T Iwasaki T Michishita I. Effects of statin therapy on plasma proprotein convertase subtilisin/kexin type 9 and sortilin levels in statin-naive patients with coronary artery disease. J Atheroscler Thromb. (2016) 23:848–56. 10.5551/jat.33407 26797266 PMC7399269

[30] Nissen L Winther S Isaksen C Ejlersen JA Brix L Urbonaviciene G. Danish study of non-invasive testing in coronary artery disease (Dan-NICAD): study protocol for a randomised controlled trial. Trials. (2016) 17:262. 10.1186/s13063-016-1388-z 27225018 PMC4880871

[31] Nissen L Winther S Westra J Ejlersen JA Isaksen C Rossi A. Diagnosing coronary artery disease after a positive coronary computed tomography angiography: the Dan-NICAD open label, parallel, head to head, randomized controlled diagnostic accuracy trial of cardiovascular magnetic resonance and myocardial perfusions. Eur Heart J Cardiovasc Imaging. (2018) 19:369–77. 10.1093/ehjci/jex342 29447342

[32] Petersen CM Nielsen MS Jacobsen C Tauris J Jacobsen L Gliemann J. Propeptide cleavage conditions sortilin/neurotensin receptor-3 for ligand binding. EMBO J. (1999) 18:595–604. 10.1093/emboj/18.3.595 9927419 PMC1171152

[33] Chang CC Chow CC Tellier LC Vattikuti S Purcell SM Lee JJ. Second-generation PLINK: rising to the challenge of larger and richer datasets. Gigascience. (2015) 4:7. 10.1186/s13742-015-0047-8 25722852 PMC4342193

[34] Marees AT de Kluiver H Stringer S Vorspan F Curis E Marie-Claire C. A tutorial on conducting genome-wide association studies: quality control and statistical analysis. Int J Methods Psychiatr Res. (2018) 27:e1608. 10.1002/mpr.1608 29484742 PMC6001694

[35] Das S Forer L Schönherr S Sidore C Locke AE Kwong A. Next-generation genotype imputation service and methods. Nat Genet. (2016) 48:1284–7. 10.1038/ng.3656 27571263 PMC5157836

[36] Jónsson H Sulem P Kehr B Kristmundsdottir S Zink F Hjartarson E. Data descriptor: Whole genome characterization of sequence diversity of 15,220 Icelanders. Sci Data. (2017) 4:170115. 10.1038/sdata.2017.115 28933420 PMC5607473

[37] Rohde PD Fourie Sørensen I Sørensen P. qgg: an R package for large-scale quantitative genetic analyses. Bioinformatics. (2020) 36:2614–5. 10.1093/bioinformatics/btz955 31883004

[38] VanRaden PM. Efficient methods to compute genomic predictions. J Dairy Sci. (2008) 91:4414–23. 10.3168/jds.2007-0980 18946147

[39] de los Campos G Vazquez AI Fernando R Klimentidis YC Sorensen D. Prediction of complex human traits using the genomic best linear unbiased predictor. PLoS Genet. (2013) 9:e1003608. 10.1371/journal.pgen.1003608 23874214 PMC3708840

[40] Athanasiadis G Cheng JY Vilhjálmsson BJ Jørgensen FG Als TD Le Hellard S. Nationwide genomic study in Denmark reveals remarkable population homogeneity. Genetics. (2016) 204:711–22. 10.1534/genetics.116.189241 27535931 PMC5068857

[41] Gilly A Suveges D Kuchenbaecker K Pollard M Southam L Hatzikotoulas K. Cohort-wide deep whole genome sequencing and the allelic architecture of complex traits. Nat Commun. (2018) 9:4674. 10.1038/s41467-018-07070-8 30405126 PMC6220258

[42] Chen H Huffman JE Brody JA Wang C Lee S Li Z. Efficient variant set mixed model association tests for continuous and binary traits in large-scale whole-genome sequencing studies. Am J Hum Genet. (2019) 104:260–74. 10.1101/395046 30639324 PMC6372261

[43] Yang J Lee SH Goddard ME Visscher PM. GCTA: A tool for genome-wide complex trait analysis. Am J Hum Genet. (2011) 88:76–82. 10.1016/j.ajhg.2010.11.011 21167468 PMC3014363

[44] Schmidt SE Winther S. cadptp: Statistical module for calculating Clinical likelihood of coronary artery disease. Int J Cardiovasc Imaging. (2019) 35:2019–2028. 10.1007/s10554-019-01662-1 31273633 PMC6805823

[45] Winther S Schmidt SE Mayrhofer T Bøtker HE Hoffmann U Douglas PS. Incorporating coronary calcification into pre-test assessment of the likelihood of coronary artery disease. J Am Coll Cardiol. (2020) 76:2421–32. 10.1016/j.jacc.2020.09.585 33213720

[46] Willer CJ Schmidt EM Sengupta S Peloso GM Gustafsson S Kanoni S. Discovery and refinement of loci associated with lipid levels. Nat Genet. (2013) 45:1274–85. 10.1038/ng.2797 24097068 PMC3838666

[47] Raffield LM Dang H Pratte KA Jacobson S Gillenwater LA Ampleford E. Comparison of proteomic assessment methods in multiple cohort studies. Proteomics. (2020) 20:e1900278. 10.1002/pmic.201900278 32386347 PMC7425176

[48] Mussbacher M Schrottmaier WC Salzmann M Brostjan C Schmid JA Starlinger P. Optimized plasma preparation is essential to monitor platelet-stored molecules in humans. PLoS ONE. (2017) 12:e018892. 10.1371/journal.pone.0188921 29220362 PMC5722331

[49] Hermey G Sjøgaard SS Petersen CM Nykjær A Gliemann J. Tumour necrosis factor α-converting enzyme mediates ectodomain shedding of Vps10p-domain receptor family members. Biochem J. (2006) 395:285–93. 10.1042/BJ20051364 16393139 PMC1422770

[50] Petersen CM Nielsent MS Nykjaer A Jacobsen L Tommerup N Rasmussen HH. Molecular identification of a novel candidate sorting receptor purified from human brain by receptor-associated protein affinity chromatography. J Biol Chem. (1997) 272:3599–605. 10.1074/jbc.272.6.3599 9013611

[51] Vaegter CB Jansen P Fjorback AW Glerup S Skeldal S Kjolby M. Sortilin associates with Trk receptors to enhance anterograde transport and neurotrophin signaling. Nat Neurosci. (2011) 14:54–63. 10.1038/nn.2689 21102451 PMC3808973

[52] Gilly A Park YC Png G Barysenka A Fischer I Bjørnland T. Whole-genome sequencing analysis of the cardiometabolic proteome. Nat Commun. (2020) 11:6336. 10.1038/s41467-020-20079-2 33303764 PMC7729872

[53] Bretherick AD Canela-Xandri O Joshi PK Clark DW Rawlik K Boutin TS. Linking protein to phenotype with Mendelian Randomization detects 38 proteins with causal roles in human diseases and traits. PLoS Genet. (2020) 16:e1008785. 10.1371/journal.pgen.1008785 32628676 PMC7337286

[54] Deming Y Xia J Cai Y Lord J Del-Aguila JL Fernandez MV. Genetic studies of plasma analytes identify novel potential biomarkers for several complex traits. Sci Rep. (2016) 6:18092. 10.1038/srep18092 PMC4698720 36647296

[55] Sun BB Maranville JC Peters JE Stacey D Staley JR Blackshaw J. Genomic atlas of the human plasma proteome. Nature. (2018) 558:73–9. 10.1038/s41586-018-0175-2 29875488 PMC6697541

[56] Kaddai V Jager J Gonzalez T Najem-Lendom R Bonnafous S Tran A. Involvement of TNF-α in abnormal adipocyte and muscle sortilin expression in obese mice and humans. Diabetologia. (2009) 52:932–40. 10.1007/s00125-009-1273-3 19219422

[57] Blondeau N Béraud-Dufour S Lebrun P Hivelin C Coppola T. Sortilin in glucose homeostasis: from accessory protein to key player? Front Pharmacol. (2019) 9:1561. 10.3389/fphar.2018.01561 30697159 PMC6340931

[58] Morris NJ Ross SA Lane WS Moestrup SK Petersen CM Keller SR. Sortilin is the major 110-kDa protein in GLUT4 vesicles from adipocytes. J Biol Chem. (1998) 273:3582–7. 10.1074/jbc.273.6.3582 9452485

[59] Winther S Nissen L Westra J Schmidt SE Bouteldja N Knudsen LL. Pre-test probability prediction in patients with a low to intermediate probability of coronary artery disease: a prospective study with a fractional flow reserve endpoint. Eur Heart J Cardiovasc Imaging. (2019) 20:1208–18. 10.1093/ehjci/jez058 31083725

[60] Riveros-Mckay Aguilera F Weale M Moore R Selzam S Krapohl E Sivley RM. An integrated polygenic and clinical risk tool enhances coronary artery disease prediction. (2020). 10.1101/2020.06.01.20119297 33651632 PMC8284388

[61] Gu F Chen TH Pfeiffer RM Fargnoli MC Calista D Ghiorzo P. Combining common genetic variants and non-genetic risk factors to predict risk of cutaneous melanoma. Hum Mol Genet. (2018) 27:4145–56. 10.1093/hmg/ddy282 30060076 PMC6240742

[62] Meisner A Kundu P Zhang YD Lan L V Kim S Ghandwani D. Combined utility of 25 disease and risk factor polygenic risk scores for stratifying risk of all-cause mortality. Am J Hum Genet. (2020) 107:418–31. 10.1016/j.ajhg.2020.07.002 32758451 PMC7477009

[63] Bom MJ Levin E Driessen RS Danad I Van Kuijk CC van Rossum AC. Predictive value of targeted proteomics for coronary plaque morphology in patients with suspected coronary artery disease. EBioMedicine. (2019) 39:109–17. 10.1016/j.ebiom.2018.12.033 30587458 PMC6355456

