# Supplementary material for: Sortilin as a Biomarker for Cardiovascular Disease Revisited
Source: Front Cardiovasc Med. 2021 Apr 16;8:652584. doi: 10.3389/fcvm.2021.652584 (PMC8085299; doi:10.3389/fcvm.2021.652584)
Supplement: Supplementary file 1 [file Data_Sheet_1.PDF]

Supplementary Material for

Sortilin as biomarker for cardiovascular disease revisited

Peter Loof Møller[1], Palle Duun Rohde[2], Simon Winther[3], Peter Breining[1,4], Louise Nissen[3], Anders Nykjaer[1,4], Morten Bøttcher[3], Mette Nyegaard[1,†,*] & Mads Kjolby[1,4,5,6,†]

[1]Department of Biomedicine, Aarhus University, Aarhus, Denmark

[2]Department of Chemistry and Bioscience, Aalborg University, Aalborg, Denmark

[3]Department of Cardiology, Gødstrup Hospital, NIDO|Denmark, Herning, Denmark

[4]PROMEMO and DANDRITE, Aarhus University, Aarhus, Denmark

[5]Department of Clinical Pharmacology, Aarhus University Hospital, Aarhus, Denmark

[6]Steno Diabetes Center Aarhus, Aarhus University Hospital, Aarhus, Denmark

[†] These authors jointly directed this work

[*] Correspondence: Mette Nyegaard, Nyegaard@biomed.au.dk

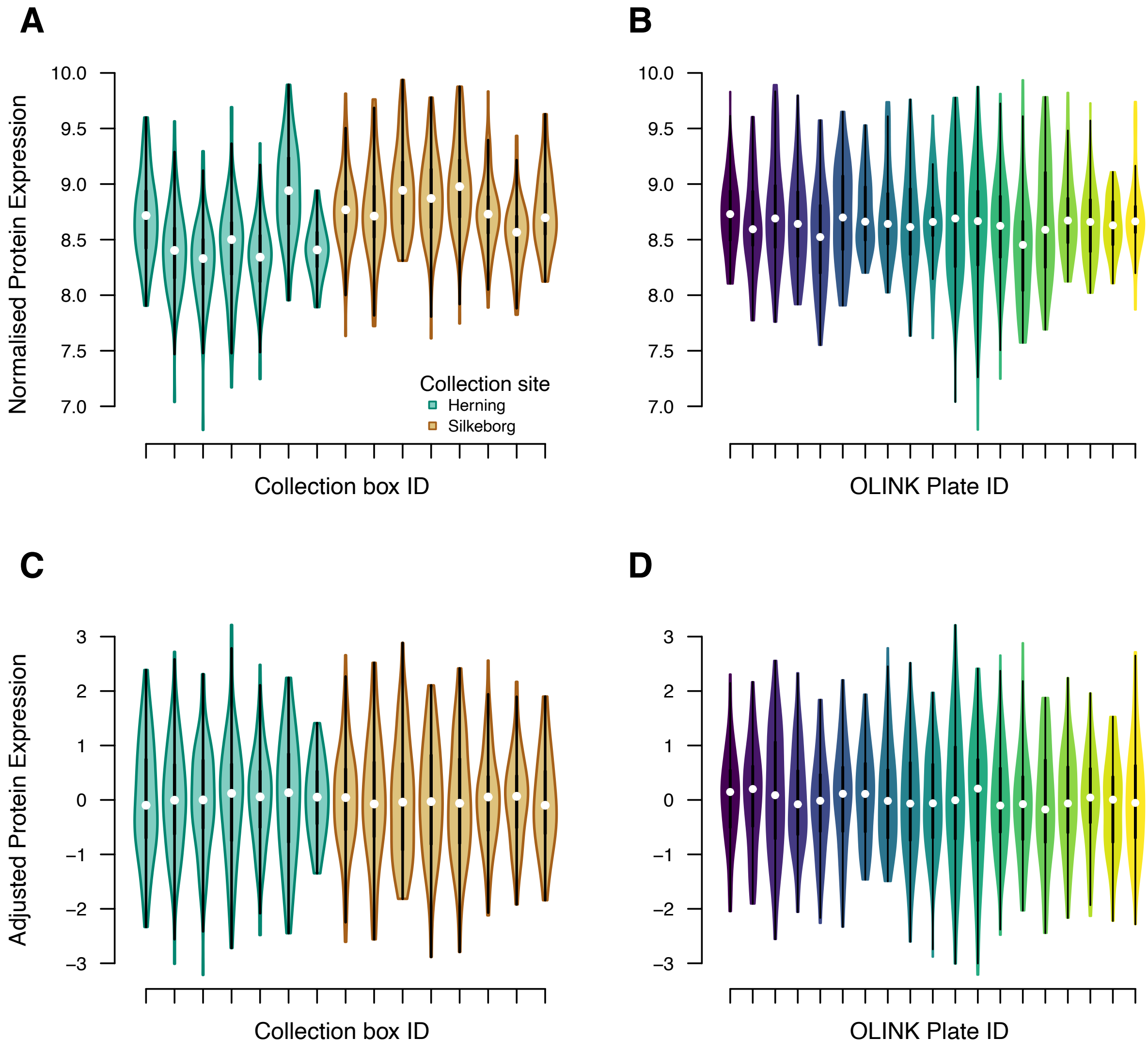

Supplementary Figure S1. A) Median normalized protein expression within collection box separated by collection site. **B**) Median normalized protein expression within OLINK plates (samples within OLINK plates were randomized). **C-D**) Median protein expression adjusted for collection box ID and OLINK plate IDs shown within collection box, and OLINK plates, respectively.

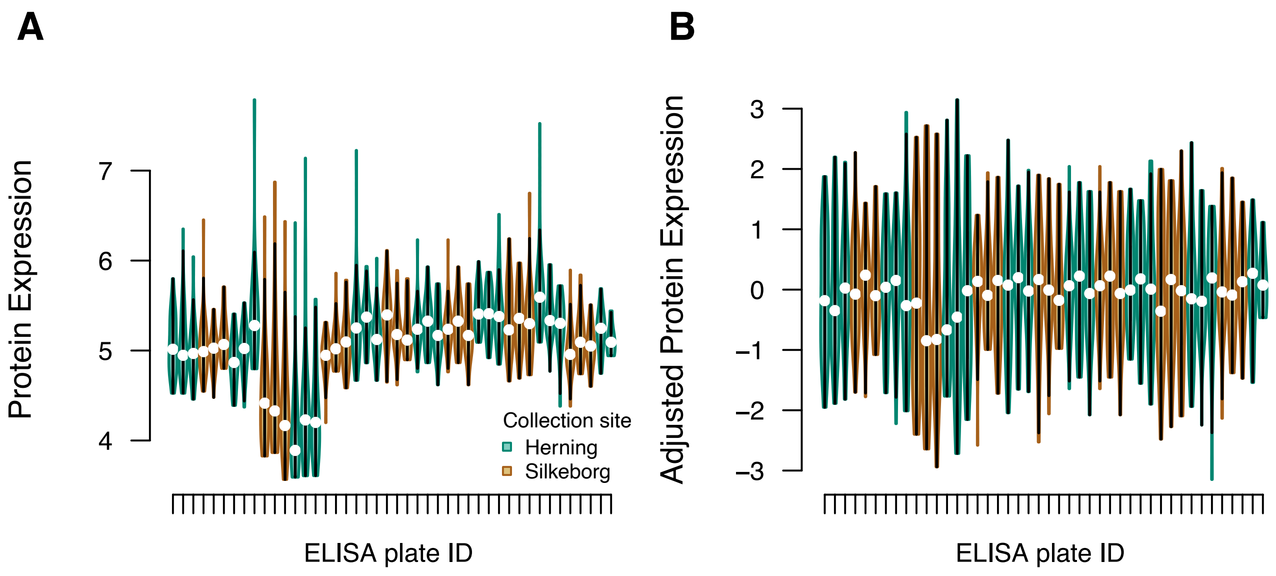

Supplementary Figure S2. A) Median protein expression within ELISA plates colored by collection site. **B**) Median adjusted protein expression within ELISA plates.

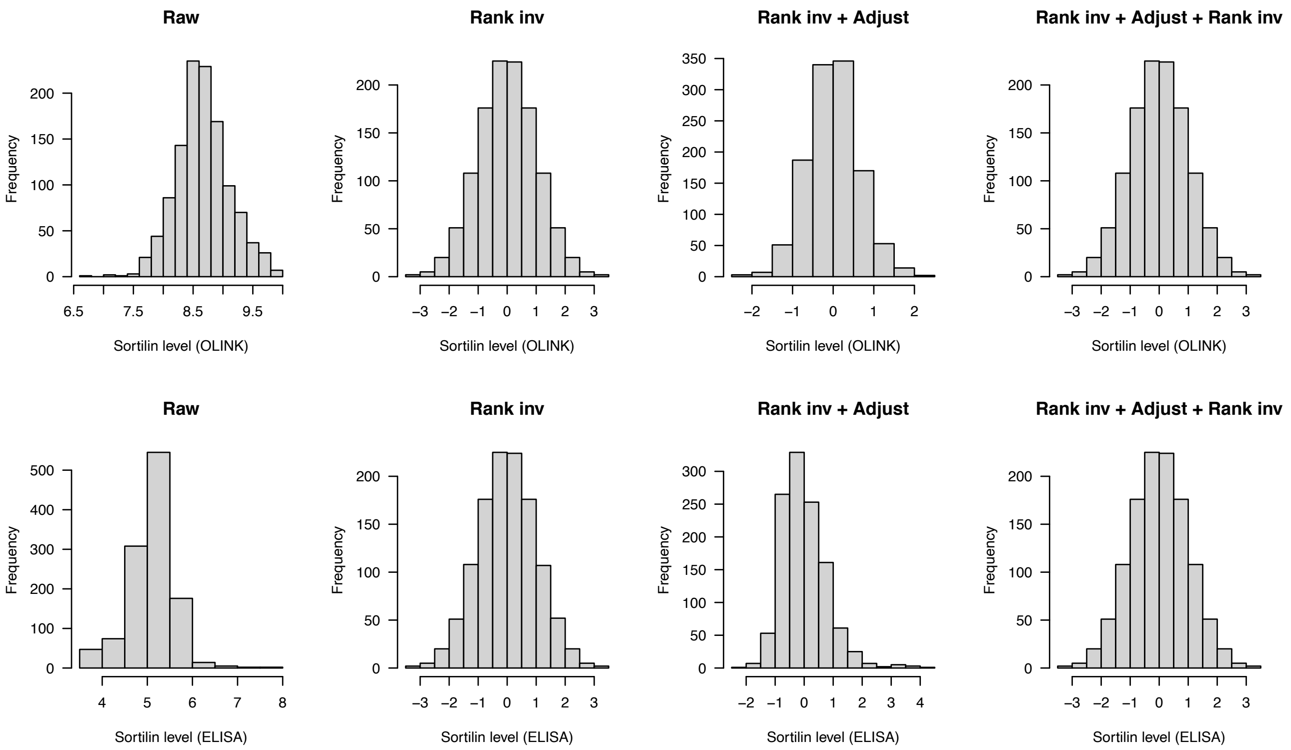

Supplementary Figure S3. Distribution of sortilin levels by OLINK (top row) and ELISA (bottom row) at the different levels of adjustment. First, the raw protein levels were rank inversed, then adjusted for covariates (sex, age, age^2, collection box ID, average protein level for OLINK CVD panel II and III [only for OLINK], and ELISA plate ID [only for ELISA]), and then the adjusted values were again rank inversed.

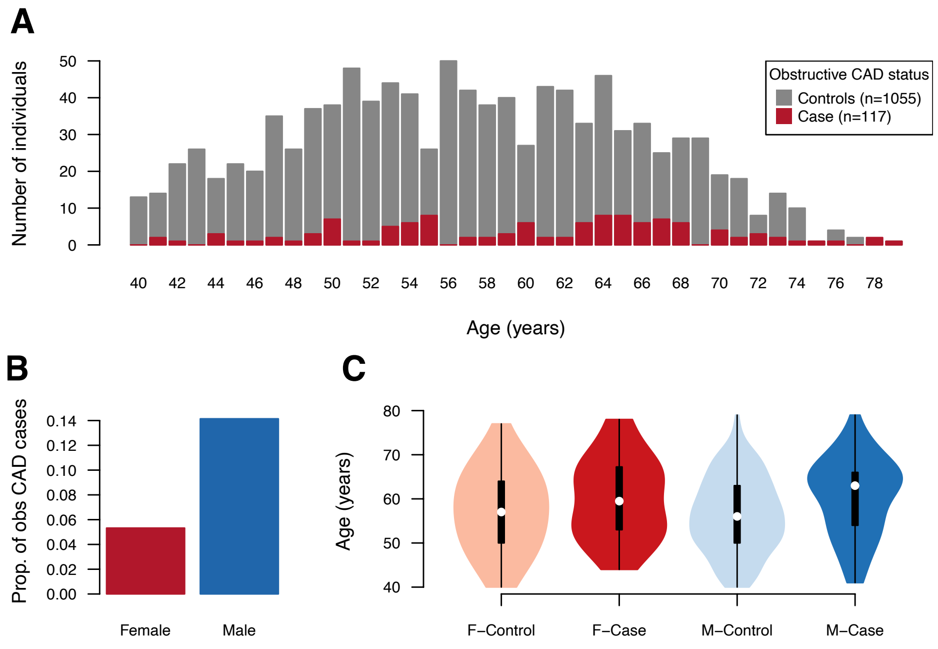

Supplementary Figure S4. A) Age distribution among individuals in the cohort stratified by obstructive CAD status. **B**) Proportion of obstructive CAD cases among female and males. **C**) Age distribution of controls and obstructive CAD cases.

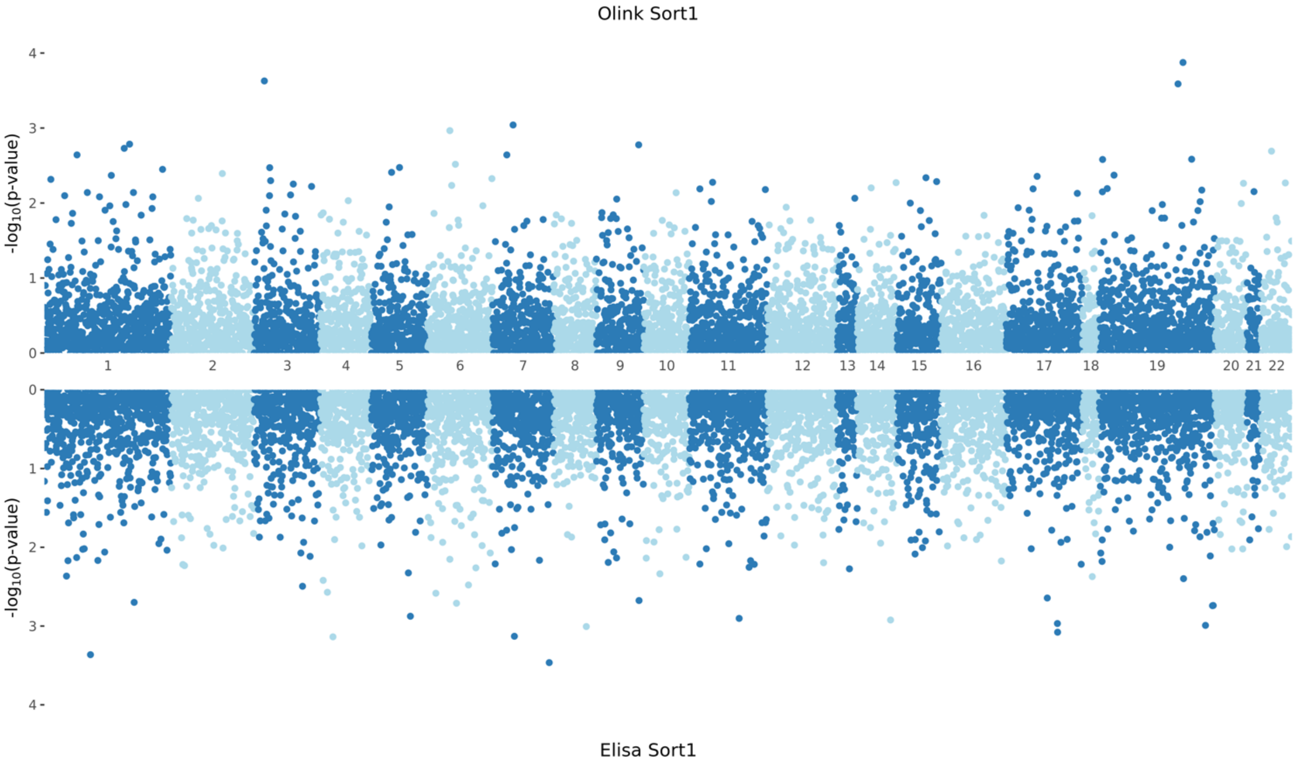

Supplementary Figure S5. Protein quantitative trait loci for variation in sortilin level quantified with OLINK (top panel) and ELISA (bottom panel). The x-axis is 7,003 genes containing variants with more severe than missense consequence from Variant Effect Predictor arranged by chromosomal position, and the y-axes show the negative logarithm base-10 to the *P*-values from SKAT-O. No signals reached genome-wide significance.

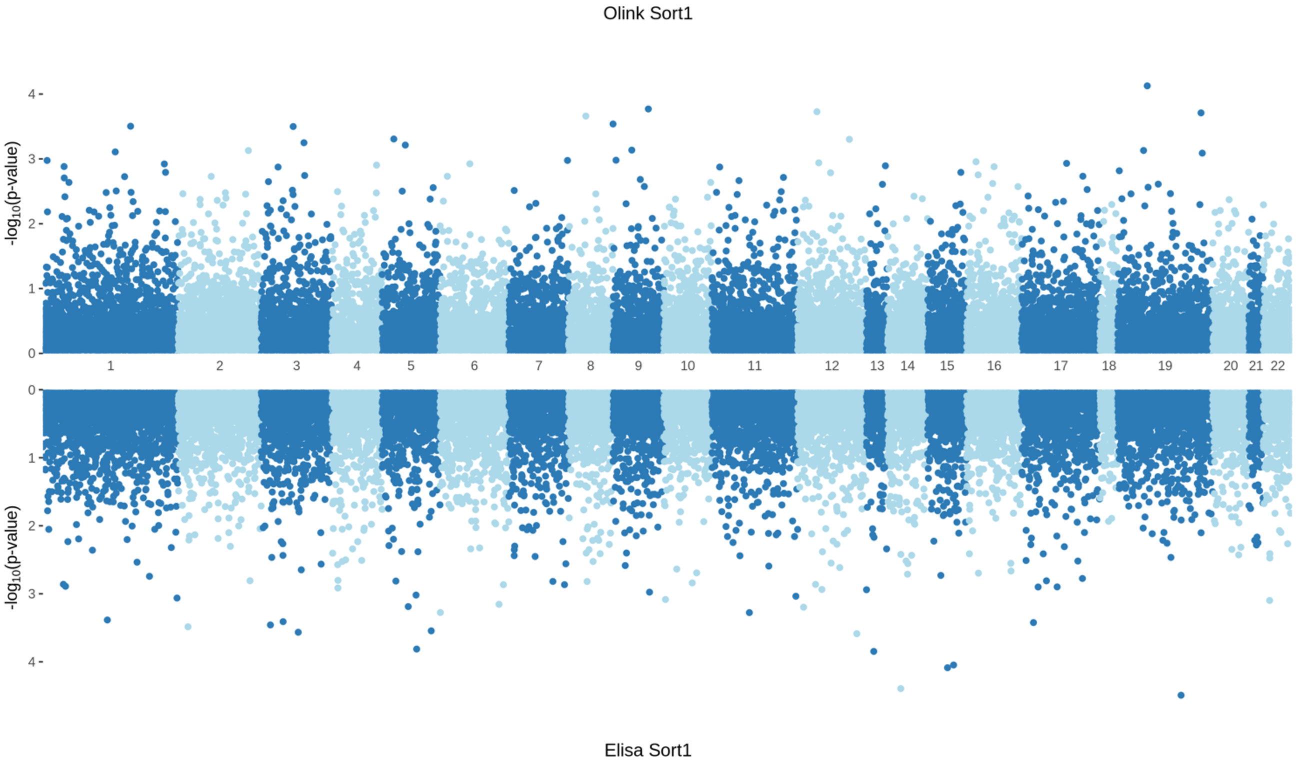

Supplementary Figure S6. Protein quantitative trait loci for variation in sortilin level quantified with OLINK (top panel) and ELISA (bottom panel). The x-axis is 17,913 genes with coding variants weighted by CADD score arranged by chromosomal position, and the y-axes show the negative logarithm base-10 to the *P*-values from SKAT-O. No signals reached genome-wide significance.
